# Rethinking the economic costs of malaria at the household level: Evidence from applying a new analytical framework in rural Kenya

**DOI:** 10.1186/1475-2875-5-76

**Published:** 2006-08-30

**Authors:** Jane M Chuma, Michael Thiede, Catherine S Molyneux

**Affiliations:** 1 Kenya Medical Research Institute (KEMRI), P.O Box 230, Kilifi, Kenya; 2 Health Economics Unit, University of Cape Town, Observatory 7925, Cape Town, South Africa; 3 Centre for Tropical Medicine, University of Oxford, Oxford, 0X3 9DU, UK

## Abstract

**Background:**

Malaria imposes significant costs on households and the poor are disproportionately affected. However, cost data are often from quantitative surveys with a fixed recall period. They do not capture costs that unfold slowly over time, or seasonal variations. Few studies investigate the different pathways through which malaria contributes towards poverty. In this paper, a framework indicating the complex links between malaria, poverty and vulnerability at the household level is developed and applied using data from rural Kenya.

**Methods:**

Cross-sectional surveys in a wet and dry season provide data on treatment-seeking, cost-burdens and coping strategies (n = 294 and n = 285 households respectively). 15 case study households purposively selected from the survey and followed for one year provide in-depth qualitative information on the links between malaria, vulnerability and poverty.

**Results:**

Mean direct cost burdens were 7.1% and 5.9% of total household expenditure in the wet and dry seasons respectively. Case study data revealed no clear relationship between cost burdens and vulnerability status at the end of the year. Most important was household vulnerability status at the outset. Households reporting major malaria episodes and other shocks prior to the study descended further into poverty over the year. Wealthier households were better able to cope.

**Conclusion:**

The impacts of malaria on household economic status unfold slowly over time. Coping strategies adopted can have negative implications, influencing household ability to withstand malaria and other contingencies in future. To protect the poor and vulnerable, malaria control policies need to be integrated into development and poverty reduction programmes.

## Background

### The economic burden of malaria

Malaria is commonly referred to as a disease of poverty and is mainly found in the poorest regions of the world [1,2]. Macro-level studies estimate that the per capita GDP in highly endemic regions is on average one-fifth that of non-endemic countries and that annual growth rates in malaria endemic countries are 1.3 percentage points lower than those of non-endemic countries, even after controlling for other factors known to influence economic growth, such as human capital and initial income [1-3]. Malaria is thought to contribute towards national poverty through its impact on foreign direct investment, tourism, labor productivity and trade. At the micro-level, malaria may cause poverty through spending on health care, income losses and premature deaths. Poor people are considered to be at particular risk of being infected because they are less likely to purchase preventive measures and to seek prompt effective treatment [2,4-6].

Although a vicious cycle between malaria and poverty is acknowledged, there is no detailed evidence around how malaria and poverty relate at the household level. Studies focus on estimating direct costs of treatment and prevention (including transport to treatment source and special foods), and the indirect costs of time lost by the sick individual and the caretaker and premature mortality [7-13]. Direct costs of malaria range from $0.41 in Malawi to $7.38 in Ghana (Table 1) [14,15]. The few studies that express costs as a proportion of household income, estimate mean direct costs from 2.0% to 2.9% [8,11,16]. These figures are well below the 10% or more of total income often taken to be indicative of costs potentially catastrophic for households [17]. Only two of the studies reviewed compare how cost burdens vary by socio-economic status. These studies suggest that costs of malaria are highly regressive; i.e. the poor spend a significantly higher proportion of their income on malaria than their least poor counterparts [8,16]. In Malawi for example, total cost burdens averaged 7.2% of monthly household income but the poor incurred an average cost burden of 32% [16].

**Table 1:** Summary of the direct costs of malaria.

Country and Authors	Direct costs per capita per month (1999 US$)		Monthly total direct costs	Direct costs as % of income
	Prevention	Treatment		
Sri Lanka [8]	-	1.91	1.91	2.0
Malawi [16]	0.05	0.41	0.46	2.0
Tanzania [42]	0.76	-	-	-
Zaire [47]	0.97	-	-	-
Cameroon [45]	1.29	2.05	3.34	-
Cameroon [41]	1.74	2.67	4.41	-
Cameroon [41]	2.10	3.88	5.98	-
Burkina Faso [44]	0.09	-	-	-
Burkina Faso [44]	0.93	1.18	2.11	-
Ghana [7]	-	0.65	0.65	-
Nigeria [11]	-	1.84	1.84	2.9

Source: Chima et al. 2003; Russell 2004

Micro-level studies provide useful information of the extent of the economic burden. However, they are based on quantitative surveys conducted on a two-week or monthly recall basis. This static approach does not capture costs that spread beyond the recall period, costs that unfold slowly over time, or seasonal variations in the burdens. The latter is particularly critical for a disease like malaria where transmission levels vary over time. Furthermore, few studies, if any, have systematically investigated the different pathways through which malaria contributes towards poverty and how poverty influences the risk of infection. The main aim of this paper is to develop and apply a framework that incorporates the range of factors consider in exploring the links between malaria, poverty and vulnerability at the household level.

### Malaria, poverty and vulnerability at the household level: an analytical framework

Developing a framework to explore the link between malaria, poverty and vulnerability at the micro-level requires some clarity on the definitions of poverty and vulnerability. Poverty is a multidimensional concept, with social, economic, cultural and political components [18]. Approaches to measurement of poverty at the individual or household level range from focusing on one indicator (for example, income or education), through combining elements to form a score (for example, asset and wealth indices), to more qualitative measures that look at poverty from the community's perspective using participatory techniques. The most comprehensive approaches consider all assets required to meet basic needs (typically broken down into human, social, financial, physical and natural assets) [18-20].

Vulnerability is closely related to poverty. However, vulnerability is dynamic and captures processes of change, while poverty is a static concept describing a situation at a fixed point in time [21]. Chambers refers to vulnerability as "the exposure to contingencies and stress and difficulty in coping with them. Vulnerability has thus two sides: an external side of risk, shocks and stress to which an individual or household is subjected, and an internal side which is defenseless, meaning a lack of means to cope without damaging loss" [22]. Poor households are usually the most vulnerable to any type of risks, but not all vulnerable households are poor. Wealthy households can be vulnerable if their resources are directed towards meeting needs that do not contribute to development and sustainability of livelihoods [23,19]. Bates et al. have considered vulnerability specifically in relation to infectious diseases [24]. They consider vulnerability as the product of a set of processes and factors operating at the individual, household/community and meso/macro level to increase an individual or group's probability of experiencing a reduction of well-being. The processes and factors they consider range from biological to institutional. In this paper vulnerability is defined as the factors that increase risk of households being subjected to the economic costs of malaria. Three dimensions of vulnerability are considered: the factors that influence cost levels, factors that determine the ability to cope with financial and time costs, and factors influencing ability recover.

**Figure 1:**
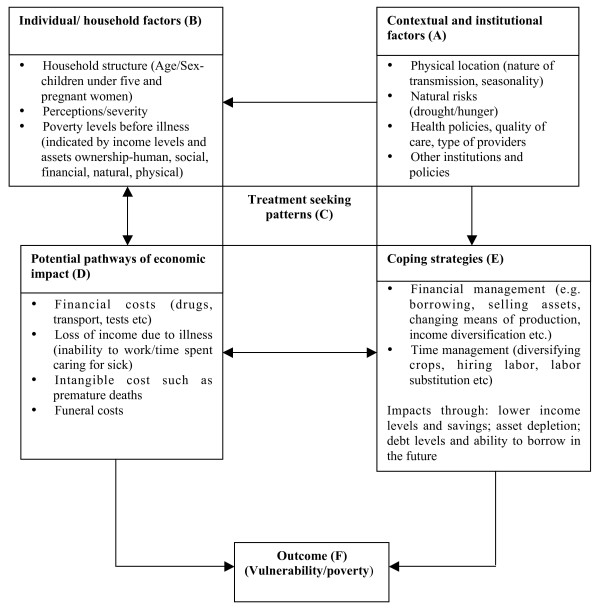
Framework for analyzing the relationship between malaria, poverty and vulnerability.

The literature around treatment-seeking, costs and coping have been drawn upon in this paper to develop a specific framework for analyzing the pathways through which malaria affects poverty and vulnerability at the household level over time (Figure 1). Contextual factors (Box A of Figure 1) determine the nature of malaria transmission in different settings, influencing who is most at risk of infection and when. In areas with stable transmission, young children and pregnant women are more susceptible to the disease, including complicated forms [25]. Vulnerability to infection may differ seasonally, with higher levels of malaria in the wet than in dry seasons. Age, gender and seasonality are therefore important determinants of malaria. The perceived nature of the illness, socio-economic status, and the household's asset base determines whether people seek treatment and the type of responses they adopt (Box C of Figure 1). The nature of the health care system, such as health policies and quality of care is also critical to treatment seeking (Box A of Figure 1).

Malaria affects households directly through spending on treatment, and indirectly through income losses by the sick persons and their carers (Box D of Figure 1). The nature of the impact incurred is determined by contextual factors (Box A of Figure 1), household level factors (Box B of Figure 1) and treatment responses (Box C of Figure 1). The premature death of a household member can pose significant costs such as transporting the body and funeral expenses [7]. Households adopt coping strategies to meet costs when they arise (Box E of Figure 1). The choice of strategy will depend on a household's asset base and the ability to transform assets into cash. Wealthy households cope better than poorer households because their assets can buffer against illness costs [26,27]. When costs are minimal, households may be able to cope by making temporary adjustments to their expenditures, other times coping strategies adopted can also have negative implications for households. The outcome can be increasing levels of vulnerability and poverty for households unable to cope with the costs of malaria and the cycle continues.

## Materials and methods

### Study setting

As in other parts of Sub-Saharan Africa, poverty is widespread in Kenya. Estimated 56.8% of all Kenyans live below the national poverty line, set as Kenya Shillings (KES) 1239 (US$ 16.5) in rural areas and KES2648 (US$ 35.3) in urban areas [28]. Poverty levels have been increasing over time. For example, in Coast province the proportion of households below the poverty line increased from 43.5% in 1992 to 55.63% in 1994 and to 62.0% in 2000.

Malaria is a major public health problem in Kenya. It affects 20 million individuals annually, kills 72 children each day and accounts for 30% of all outpatient visits in the country [29,30]. The financing of the health sector has been undergoing reforms since the 1970s. Key changes include introducing user fees in government facilities and a reduction of health budgets. The annual health care expenditure declined from 9.3% of GDP in the 1980s to 5.1% in the 2001/2002 financial year [31]. Total per capita health expenditure is KES 1440 (US$ 19.2) per year, a figure well below the World Health Organization (WHO) recommendation of KES 2550 (US$ 34) [32]. About 51% of total health care expenditure comes from households through out-of-pocket payments [31]. Although malaria is a major cause of morbidity and mortality, the actual costs borne by households and how such costs hinder economic development are not well documented. An improved understanding of the economic impact of the disease may justify additional efforts and resources directed towards malaria control activities.

The study was conducted from January 2003 to October 2004 in Ganze, in Kilifi district. Kilifi district is the second poorest in Kenya with the highest female illiteracy rates in the country. An estimated 64% of Kilifi residents cannot afford to meet the minimum food requirements, even if they were to spend all their income on food alone [33]. Ganze location is hot and humid. Malaria transmission is low and stable, with peak seasons between April and July [30]. There is one government dispensary serving a population of 35,299 persons, the next closest government facilities are a health center located twenty kilometres away and Kilifi district hospital 35 kilometres away. Other providers in the area include two private clinics, numerous shops selling over-the-counter drugs and traditional healers. The dispensary and private clinics are situated at the Ganze trading centre. Given that the majority of the population live in the interior, a long walk or bike ride are needed to reach these services.

### Data collection and analysis

The data presented in this paper come from a broader study that investigated the impact of costs of all illnesses on household livelihoods in both a rural and an urban setting. This paper focuses on the links between malaria, poverty and vulnerability in the rural setting. As with many treatment-seeking studies [7,8,13,26,27,34], a limitation of this study is the use of reported fever as the main indicator of malaria.

The study was conducted in two main phases:

• Household surveys: Maps indicating the location of every homestead and landmarks were drawn by hand to enable the random selection of survey households. A total of 294 households were visited in the wet season. The same households were then visited in the dry season (n = 285, 9 refusals). The survey gathered information on socio-demographic characteristics, direct and indirect costs, and expenditure and coping strategies. The questionnaire was administered to the household head or spouse and in his/her absence, another senior adult member of the household. The majority of the respondents were female since males were often absent. For illnesses reported among adults and children, efforts were made to interview the ill person and the primary carer respectively.

• Case studies: 15 households were purposively selected to represent varying degrees of vulnerability and poverty in the community and thereby contribute to deepening theoretical understanding. Selection was based on indicators of socio-economic status (including poor and less poor households), cost burdens (including households with high and low costs) and coping strategies (Figure 1). Case study households were visited monthly for a year. Data on illness, treatment seeking patterns and cost burdens were collected once every month and updated until full recovery was reported. More in-depth information on other aspects highlighted in Figure 1 were captured in an additional five sets of visits organized over the follow-up period. Topics included factors that influenced the household's situation before the study started, the range of assets households have access to, social networks and their role in meeting malaria costs, and household debt and repayment. A final visit at the end of the study explored changes in asset composition over the study period, and perceived reasons for that change.

Analysis of both qualitative and quantitative data was organized around the key variables at different levels in the framework. Poverty among survey households was estimated using expenditure as a proxy for income. Households were allocated to different Socio-Economic Status (SES) categories using data on expenditure as proxy for income. Expenditure data were converted into per capita estimates for each household weighted for age using adult equivalence scales. [35]. Direct costs were classified as all cash spending due to malaria for both the patient and caretakers. These included spending on consultation, drugs, tests, gifts, transport, special foods and any other costs that a household incurred due to illness. Indirect costs were measured in terms of the number of days that the ill person and their caretakers were unable to conduct their activities due to illness. Income days lost were valued in monetary terms by using an average daily income estimated from the surveys. Days lost among case study households were valued in monetary terms only if the illness translated into actual income losses. Total costs were estimated by summing up direct and indirect costs.

Recorded qualitative data were analyzed manually using content analysis [36] to identify common themes and sub-themes. Case study households were reclassified into vulnerability and poverty groups using information collected in the first two visits, including number of workers, types of jobs, types and severity of shocks experienced five years prior to the study, their impact, and apparent ability to cope and recover from negative impacts. Case study households were then re-classified at the end of the research using similar indicators. The role of malaria in any changes in category was then assessed using the data collected over the one year period.

## Results

The findings are organized around the framework, with each sub-section addressing factors highlighted in Boxes A-F of Figure 1. First a description of factors that influence vulnerability to the costs of malaria at a broader community level is presented; second an overview of treatment seeking patterns, cost burdens and coping strategies among survey households and finally the additional data emerging from the case studies, which bring together all aspects of the framework.

### Contextual and household level factors influencing vulnerability (Boxes A and B of Figure 1)

The survey provided substantial information on the socio-economic and demographic characteristics of the community. Education levels are low, with 438 out of 819 adults (53.4%) not having received any education. The main sources of income for household heads are small scale farming (n = 135; 62.2%), followed by unskilled labour such as building and construction (n = 38; 17.5%). Mean monthly per capita expenditure was KES 989 (US $12.7) in the wet season survey and KES 913 (US$ 11.7) in the dry season (p = 0.3579). The dry season is reportedly difficult for households because casual farm jobs are not available and food is scarce. Expenditure was unequally distributed among poor and less poor households; those in the wealthiest quintile spent ten times more than the poorest quintile. About 80% of survey households live below US$1 per day.

The majority of households own land (88.4%). For about four years, crop yields have been low due to rain shortages. The year that the case study took place (2003–2004) was reported as particularly bad, with people living 'hand to mouth'. Since land and casual labor are critical income generating sources drought limited people's access to cash and made their livelihoods vulnerable. Attempts to diversify income sources (for example through small businesses, selling local brew, or weaving) were often futile because the market for such goods was constrained by cash flow problems across the community. An important safety net is having livestock, which can be transformed into cash when the need arises, thereby acting as a form of bank. The survey revealed the main types of livestock are chickens (256; 87.1% households) and goats (198; 67.3%). A minority of households own cows (57; 19.5%). However, the market for livestock was also constrained by the drought: people found it difficult to sell livestock, and animals fetched much lower prices than they would normally. Other key assets owned by households were radios, bicycles and sewing machines (46%, 20%, and 10% respectively).

### Treatment seeking behavior, economic pathways and coping strategies (Boxes C-E of figure 1; survey data)

Table 2 summarizes the main treatment-seeking findings from the wet and dry season surveys. As would be expected given malaria transmission patterns, the proportion of households reporting malaria was significantly higher in the wet than in the dry season (64% and 37% respectively; P < 0.001). About 80% of all reported illnesses were treated. Main treatment-seeking actions were self-treatment using drugs bought from local shops in both seasons (47.9% of all actions in the wet season, 43.9% in the dry). The types of formal health care services used differed significantly between seasons; people used the government dispensary more in the dry than in the wet season (13.1% and 6.8% respectively), while the use of private clinics was higher in the wet season than in the dry season (9.5% and 5.2% respectively).

**Table 2:** Reported malaria and treatment seeking patterns among survey households.

	**Wet season (%)**	**Dry season (%)**	**P values**
Households reporting at least one malaria episode 2 wks before survey	187 (63.6)	104 (36.5)	<0.001
Number of ill individuals	307 (14.2%)	187 (8.8%)	<0.001
Individuals reporting malaria by age			
• <5	95 (31.0)	70 (37.5)	0.79
• 5–<10	62 (20.2)	36 (19.3)	0.80
• 10–<18	60 (19.5)	34 (18.2)	0.71
• 18–<35	37 (12.1)	24 (12.8)	0.78
• 35+	53 (17.3)	23 (12.3)	0.14
Actions taken within HH:			
• Herbs	26 (7.5)	19 (11.5)	0.13
• Modern drugs already there	22 (6.4)	6 (3.6)	0.21
• Prayers	18 (5.2)	9 (5.5)	0.89
Actions taken outside HH:			
• Shops	196 (56.7)	84 (51.2)	0.25
• Private clinic	39 (11.3)	10 (6.1)	0.06
• Government	30 (8.7)	25 (15.2)	0.00
• Healer	3 (0.9)	3 (1.8)	0.39
	12 (3.5)	8 (4.8)	0.47

Table 3 summarizes the direct and indirect cost burdens and the strategies adopted by households to cope with these costs. Mean monthly direct costs were higher in the wet season (7.1%) than in the dry season (5.9%), although not significantly. Direct cost burdens were regressive with the poorest households spending over 10% of their expenditure in both seasons. There was an increase in mean cost burdens in the dry season among the poorest households because their income sources were particularly dependent on season. Among other categories of households cost burdens were relatively lower although these differences were not statistically significant. Indirect costs were lower than direct costs, but were reported among 54.5% of households in the wet season and 58.3% of households in the dry season.

**Table 3:** Cost burdens and coping strategies among survey households.

**Variable**	**Wet season**	**Dry season**	**p-value**
Mean monthly expenditure per household in KES (median)	271 (55)	165 (40)	0.13
Mean monthly direct costs as % of expenditure (median)	7.1 (2.1)	5.9 (1.4)	0.58
Mean monthly indirect cost as % of expenditure (median)	5.4 (0.0)	2.1 (0.0)	0.04

**Mean direct costs as % of monthly expenditure**			
• Poorest	11.0	16.1	0.47
• Very poor	7.8	3.2	0.18
• Poor	5.0	3.7	0.59
• Less poor	6.8	3.3	0.37
• Least poor	3.4	2.6	0.57

**Indirect costs as % of monthly expenditure**			
• Poorest	8.1	1.9	0.17
• Very poor	5.7	3.3	0.47
• Poor	3.4	3.5	0.89
• Less poor	1.4	1.5	0.43
• Least poor	1.6	0.5	0.9

Households adopting coping strategy (%)*	**n = 74 **	**n = 42 **	
• Borrowing	37 (50.0)	29 (69.0)	0.05
• Gifts	29 (39.2)	7 (16.7)	0.01
• Sell labour	21 (28.3)	4 (9.5)	0.02
• Sell assets	6 (8.1)	7 (16.7)	0.22
• Credit from health care provider	8 (10.8)	6 (14.3)	0.57
• Other (mixed)	14 (18.9)	12 (28.6)	0.25

* Total adds up to more than 100% because some households adopted more than one strategy.

Among households whose treatment responses required payment (n = 149 in wet season and 76 in dry), 50.7% did not have cash readily available in the wet season and 55.3% in the dry. These households adopted coping strategies such as borrowing, selling assets, selling labor among others. Although the overall proportion of households requiring some form of coping strategy did not differ significantly by season, strategies did: there was more gift giving and sale of labor in the wet season, and more borrowing with an expectation of return in the dry season.

### Exploring the links between malaria, poverty and vulnerability – case study findings

#### Vulnerability and poverty before and at the end of the research

Case study households were classified into three categories at the beginning and at the end of the study. Their characteristics are summarized below:

(a) Highly vulnerable households (n = 5) had:

• Experienced a stressful event in the past and had not yet recovered. All had depleted their assets and were descending into poverty when the research started. Three reported the main cause of their economic decline to be malaria;

• Accumulated large debts they had not been able to repay. Often these debts were accrued in their attempts to finance past treatment-seeking, especially hospitalizations and funeral expenses;

• No source of regular income, jobs were insecure and unpredictable;

• Limited asset bases and were 'struggling' to survive.

(b) Vulnerable households (n = 6) had:

• Experienced a stressful event in the past but still had assets that would enable them to cope with future costs. Two reported having had their livelihoods affected by malaria prior to the study;

• At least one source of regular income, but no permanent employment;

• A moderate asset base (all owned cows or goats).

(c) Least vulnerable households (n = 4):

• Did not report any events with major impacts on their livelihoods. None reported major malaria episodes in the years preceding the study;

• Had at least one member with a permanent source of income (thus secure income in the long-run)

• Had accumulated assets, primarily cows and goats and had savings with financial institutions.

**Figure 2:**
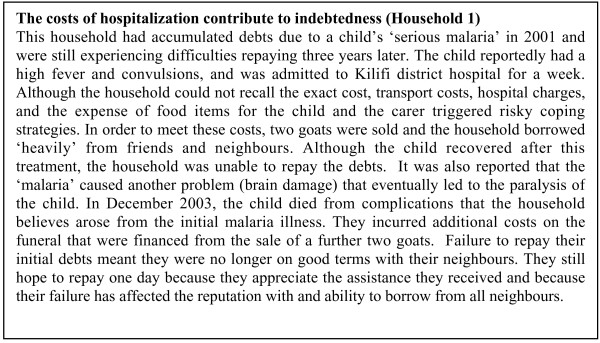
The costs of hospitalization contribute to indebtedness (Household 1).

**Figure 3:**
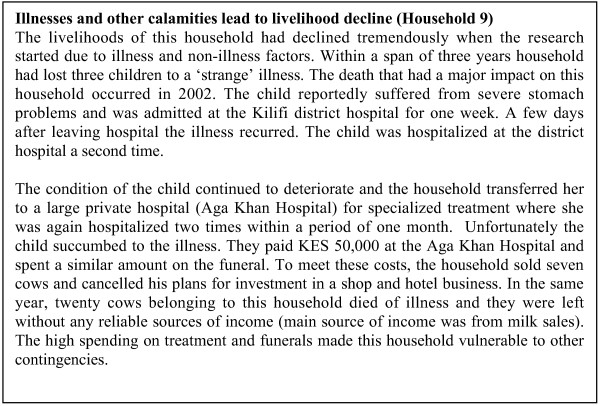
Illnesses and other calamities lead to livelihood decline (Household 9).

The pathways through which malaria imposed adverse effects on households prior to the study included high spending on hospitalization and funerals, the sale of livestock, accumulating debts that affected their ability to borrow in the future and the drought. Two cases of households that illustrate the inter-related nature of these influences are summarized in Figures 2 and 3.

At the end of the research, households were classified into three categories reflecting their outcome (Box E of Figure 1). As noted above, categorization was based on similar indicators to those used at the outset. Households were categorized as 'declining' if they recorded negative changes in two or more indicators, 'stable' if there were no changes or if a reduction in one dimension was compensated by an increase in another, and 'improving' if two or more indicators increased. All households defined as highly vulnerable at the outset declined, vulnerable households either declined, remained stable or improved, while none of the least vulnerable households declined (Table 4). Key potential factors contributing to the outcome at the end of the research were: drought, hunger and associated stresses; levels of reported malaria; and treatment seeking, payment and coping patterns

**Table 4:** The outcome at end of the research among case study households.

Status at the beginning	Status at the end of the research		
	
	Declined	Stable	Improved
Highly vulnerable	5	1	0
Vulnerable	1	4	1
Least vulnerable	0	1	3

#### Factors influencing outcome at the end of research

##### Contextual factors influencing outcome (Box A of Figure 1)

Drought and hunger had a significant impact on household outcome at the end of the research. Since most households' main source of income was farming, income levels declined tremendously, and there was barely anything to spend after food. People regularly talked about their frustrations in farming and blamed inadequate rains for all their problems:

"The big problem we face is drought, but if there is enough rain then things would change. Even the number of people getting sick would go down. You see people would eat well...food is important." (Young man, Malomani)

"In such times [when there is rain] we say 'mambo ni kwao' [everything is fine] because when there is rain, even the jobs become available. The young people get jobs. Even people can drink more when they see there is enough food in the home. This is a time to make merry." (Young man, Vilwakwe)

**Figure 4:**
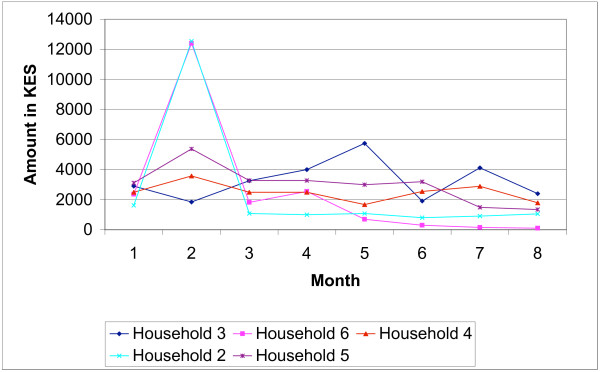
Declining levels of monthly income.

The drought affected different aspects of livelihoods. Even illnesses were left untreated, as putting food on the table was the most urgent need. As one household put it *"How can I take the child to the hospital while last night we slept hungry?"(Household 3) *The highly vulnerable households were the most affected since they did not have any source of regular income. The declining levels of income over the case study period for a selection of households are illustrated in Figure 4.

##### Self reported malaria, and treatment seeking, payment and coping patterns (Boxes B-E of Figure 1)

Table 5 shows the distribution of self-reported malaria across household and vulnerability categories, and indicates treatment-seeking and cost data. A total of 119 malaria episodes were reported among all households over 8 months, with most households reporting a mean of one episode per person over the period. Levels of reported malaria varied from month to month, with high peaks in the third and sixth months. The peak in the third month corresponded to an expected transmission increase with the wet season. The six-month peak was during a period when weekly diaries on illness and treatment seeking were being filled and collected, improving recall. As expected, households with young children reported the highest number of episodes. There were no noticeable differences in the levels of reported malaria by vulnerability category. Shops were the main source of treatment for all households. Least vulnerable households used the relatively expensive private clinics more consistently than other households, who were more likely to use the government dispensary. Herbs and healers were reported much more than they were in the surveys, and primarily among highly vulnerable households.

**Table 5:** Self reported malaria, treatment sources and direct cost burdens among case study households over 8 months.

Household	Self reported malaria		Number of times household used type of treatment					Average monthly cost burdens (%)	Outcome at end of the research
			
	Total episodes	Per capita episodes	Shops	Dispensary	Private	Herbs	Healer		
1	9	1	1	2	4	0	0	19.6	Declined
2	8	1	3	3	0	2	1	0.3	Declined
3	7	1	2	1	2	2	1	6.0	Declined
4	3	0	3	0	2	0	0	0.5	Declined
5	9	1	2	0	0	4	0	0.3	Declined
6	5	1	0	1	3	0	2	12.1	Stable
7	7	1	4	1	0	1	0	0.2	Stable
8	7	0	5	2	1	0	1	5.1	Stable
9	28	1	15	1	6	0	1	7.3	Improved
10	10	2	1	0	0	1	0	0.1	Declined
11	5	1	0	0	0	0	0	0.0	Stable
12	5	1	1	0	4	0	0	2.0	Stable
13	13	2	8	1	3	0	0	1.0	Improved
14	3	1	2	0	1	0	0	1.1	Improved
15	0	0	0	0	0	0	0	0.0	Improved
**Total**	**119**		**48**	**12**	**19**	**10**	**5**		

*Household 1–5 are the highly vulnerable, household 6–11 are the vulnerable and household 12–16 are the least vulnerable.

**Figure 5:**
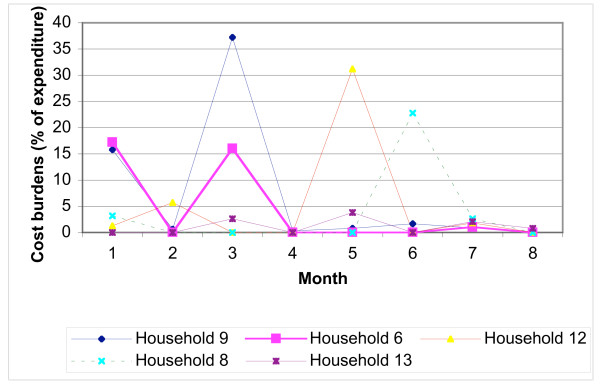
Distribution of cost burdens over 8 months.

Health expenditure per episode ranged from zero to US$ 22.82 (mean US$ 2.13). The mean cost of buying drugs from the shop was US$ 0.30 range (US$ 0.04–1.03), for visiting a private provider US$ 4.59 (range US$ 1.28–13.01), and at the government dispensary US$ 0.64 (range US$ 0.13–1.28). Most households reported mean monthly cost burdens of below 5% of total expenditure, as with the survey. However costs were not smoothly distributed over 8 months (Figure 5), with seven households having cost burdens of over 10% for at least one month over the case study period. Fluctuations depended on the number of episodes reported within the household, perceived severity of episodes and the type of treatment and coping strategies sought.

Borrowing from friends and neighbours, gifts and credit from shopkeepers and private providers were the main source of support for all households, but the way in which these and other strategies are enabled or constrained differ by vulnerability category. Highly vulnerable households had more limited support, and could only access small amounts of money (below KES 100 and often much less), network members were in a similar or worse economic situation, or they were considered too poor to be trusted with loans:

"Some people think that when they assist you, you will not be able to pay back, so they refuse to give...they tell you they do not have even when they know very well they have." (Household 3)

"I have three good neighbours, they are my friends...I see them every day in church, but their support is low because they do not have. We only help with small things...a few shillings to buy salt or flour." (Household 2)

Among some highly vulnerable households even KES 10 would require the adoption of some form of coping strategy; primarily borrowing from friends. Other coping strategies did not aim to raise cash, such as borrowing drugs from neighbours, and sharing drugs between siblings. Drugs were mainly shared among siblings who fell ill within the same period. This strategy was common among households with many young children. Usually the younger or more seriously ill child was taken to a health facility and the drugs used to treat siblings. Where raising cash or other strategies were impossible, 'ignoring' illness and struggling on was common among the highly vulnerable households. Thus, treatment seeking behavior, in often in itself a coping strategy that minimizes the amount of cash spent on treatment.

## Discussion

In this paper, literature and existing frameworks around poverty, vulnerability and treatment-seeking, costs and coping have been drawn upon to present a framework to investigate the role of malaria in vulnerability and poverty among rural households. The framework goes beyond estimating the direct and indirect costs of malaria towards identifying other economic pathways through which malaria increases household vulnerability and poverty. It has then been applied to data from a low-income setting on the Kenyan coast. The results contribute to a small body of micro-level studies that explore the links between malaria, poverty and vulnerability. In this section three sets of findings are discussed in turn: cost burdens and their seasonality; factors contributing to malaria vulnerability; and the relationship between malaria cost burdens and outcome.

### Seasonality of cost burdens

Households reported more malaria episodes in the wet than in the dry season. Direct cost burdens were higher in the wet compared to the dry season. These findings are expected: in Ganze as in many agricultural communities, the wet high transmission season is a busy period with relatively good availability of casual work and farm income. Having high levels of malaria in these 'boom' periods has important negative implications for the well-being over time. This may contribute to higher use of private clinics in the wet season, and higher use of the dispensary in the dry season. People are keen to avoid the relatively long dispensary queues in the wet season and are better able to meet relatively high private clinic costs. Indirect costs were also significantly higher in the wet than in the dry season because the opportunity costs of time were higher in the former.

The literature review did not identify any study that has looked specifically at seasonal differences in the costs of malaria. However, one study explored seasonal differences in costs of all illness in a rural setting in Burkina Faso (i.e. not malaria specific) [27] and observed the opposite pattern: more illnesses and higher costs in the dry season. Other studies have noted higher utilization of health care services in the dry season [37]. Sauerborn et al. [27] argue that since the rainy season is a busy season for agricultural communities, people have 'less' time to be ill and perceive illnesses to be less severe because they prefer to work rather than take precious time off to seek treatment.

The findings presented in this paper reflect expected seasonal differences in malaria transmission, treatment-seeking and cost burdens. There are further factors that may have contributed to these study findings. Firstly, the drought may have altered norms of production, income levels and spending patterns. Secondly, methodological differences in valuing income and costs can have an impact. Sauerborn et al. [27] valued home production to estimate income while expenditure was used in this study. Expenditure is generally preferred to income among agricultural communities because households consume a large proportion of their own production, which is difficult to value. Income is also subject to fluctuation while expenditure is smoothed over time, making it a better indicator of socio-economic status over a year [38].

The importance of incorporating seasonality into estimates of the economic burden of malaria is clear. At the very least, the timing of surveys and other contextual information such as economic activities and malaria transmission patterns should be provided to enable comparisons between studies. Regarding policy and practice, should the findings be replicated in similar settings elsewhere, lowering charges and enabling debts to be repaid in less economically challenging periods, could be considered.

### Factors contributing to malaria vulnerability

The case study findings revealed that the history of households has important implications for cost burdens, ability to cope and the outcome. Some households that had incurred high costs due to repeated malaria related deaths and hospitalizations within the five years prior to the study were highly vulnerable by the time the research started. Their situation continued to decline through out the study period. Most households incurred cost burdens of over 10% in two out of eight months. In other months costs burdens were relatively low. The concentration of costs within a few months can have implications for how costs are managed. Since costs are met at the time when they occur, mobilizing resources to finance them can affect household ability to cope with other shocks in the future.

Borrowing was the main strategy among survey households, preferred because it is relatively quick and minimizes delay in treatment. The importance of borrowing has been shown elsewhere for malaria [26,39] and general illness costs [40,27,42]. Additional information presented in this paper is around decision-making and access surrounding each strategy. These differed by household wealth and reputation. Least vulnerable households had good access to less risky strategies such as credit from local shops and private providers, while highly vulnerable households had limited choice of strategies. Coping strategies were evidently far more complex than is possible to gather in a survey. For example, borrowing from friends and neighbours was not preferred because it was prone to gossip. Social networks were also strained by drought. Highly vulnerable households were excluded or excluded themselves from borrowing networks because they were deemed too poor to pay and were unable to reciprocate. Ignoring illness and preventing costs from arising was therefore a basic survival strategy for poor and vulnerable households. Although coping strategies can be successful in meeting the costs of treatment in the short run, they can have negative implications in the long term. For example, debt accumulation and sale of livestock made households poorer and more vulnerable to other contingencies in the future. A potential way of increasing ability to cope is to build assets that cushion the poor from the costs of malaria and other shocks. This might include encouraging livelihood diversification and expanding markets for livestock products such that households have alternative sources of income to sustain them during rain shortages.

### The relationship between malaria cost burdens and outcome

The longitudinal data revealed no clear relationship between costs burdens and outcome at the end of the research. Some households with low cost burdens declined while others improved or remained stable, and visa versa. However, when cost burdens, vulnerability categories at the beginning of the research and the outcome are taken together, it is clear that all highly vulnerable households declined (irrespective of their cost burdens), vulnerable households declined or remained stable, and none of the least vulnerable households declined. This pattern can be attributed to various factors:

• Exposure to risk of infection and cost burdens does not always lead to increased vulnerability or poverty: households' assets endowments can mediate potential impact through supporting cost management strategies. Households that had a good asset base were able to meet arising costs without depleting their resources and without risking any decline;

• For poor and highly vulnerable households, the type of treatment-seeking behavior selected was often a coping strategy. Highly vulnerable households prevented costs by adopting treatment actions that did not require cash (for example herbs) and by not seeking any treatment. Therefore, low cost burdens do not necessarily imply less need but rather can indicate desperation or affordability barriers to seeking care. In a community characterized by high levels of poverty and food insecurity, seeking health care might not be 'important' because diverting even small amounts away from food towards shop drugs is difficult.

• Household poverty and vulnerability is influenced by many factors and the costs of malaria are just one. Key factors identified in this study were natural factors (drought and hunger); economic factors (types of jobs and income levels); social and demographic factors (household composition and management of resources); and illness and health (number of reported illnesses). Based on this it is difficult, if not impossible, to associate a single factor to outcome. For example, some households sold assets to buy food, pay school fees for their children or to pay for the treatment of other illnesses (not malaria). What is clear is that high costs of treatment, together with accumulated debts and sale of animals led to increased poverty and vulnerability for some households and constrained improvements for others.

Critically, it is clear that highly vulnerable households suffer and struggle on with illnesses, risking complications that might require more expensive treatment strategies. Such households urgently require protection from health care payments and their livelihoods supported through equitable economic development.

An important caveat with the above finding is the definition of malaria as reported fevers. Only 40–60% of fevers may be clinical malaria [34,51]. This limitation is difficult to overcome, given the practicality of field based testing. The implications are that clinical malaria costs may be lower; costs of uncomplicated malaria may be overestimated but the impact of severe disease may be underestimated, although this depends on what non-malaria fevers are and associated treatment seeking patterns. Nevertheless many of the above recommendations are not specific to malaria interventions, but support of general households sustainability.

## Conclusion

Understanding the various pathways through which malaria causes poverty is critical for targeting malaria control interventions towards the poor and vulnerable. By applying the framework developed in this paper the findings have contributed towards understanding the economic burden of malaria in various ways. Firstly, the study has shown that low cost burdens do not necessarily indicate less burden, rather they might indicate higher levels of need among poor households who prevent costs from arising by leaving illnesses untreated, thereby risking complications. Secondly, the costs incurred as a result of the range of coping strategies adopted are often high and have significant impact on household well being, through for example sale of assets. These impacts spread over time and determine a household ability to withstand malaria in future together with ability to cope with other contingencies. Thirdly applying the framework presented in this paper highlights the range of issues that need to be tackled to minimize the economic burden of malaria. Malaria control policies and interventions need to be integrated into sustainable development and poverty reduction initiatives. Finally, the adoption and application of the framework presented in this paper in other settings will tests its value and generalizability of the findings.

## Authors' contributions

JC was responsible for data collection, analysis and writing up of the paper and assisted CM with the design of the study. CM and MT provided support on the overall study and commented on several drafts of the manuscript. All authors read and approved the final manuscript.

**Table 6:** Summary of coping strategies reported among case study households.

Strategy	Highly vulnerable	Vulnerable	Least vulnerable
Borrowing	Rarely borrowed cash because they were not creditworthy (too poor to pay back); fear of borrowing and being unable to pay back leading to bad reputation & gossip	A common strategy because they had moderate assets but still not enough to rely more on other sources of credit like shops or private providers	Not common because they had other sources of credit but they could easily borrow if need arose.
	Amount of money borrowed was small (KES 10) because their friends were equally poor	Could borrow up to KES 100–200	Could easily borrow KES 5000 if need be because their friends were in a good economic situation
Credit from private providers	Not accessible by these households due to poverty	Could get treatment on credit but limited amounts depending on providers understanding of their economic status	Unlimited access to credit from providers because they were wealth, had permanent jobs & could easily pay by end of the month
Credit from shops	Occasionally but small amounts to buy drugs	Had access to credit but could be denied when they asked for large amounts	Could acquire all goods on credit until end of the month
Sale of assets (Goats & chickens)	Those that had assets sold them to pay for treatment or other needs but some had nothing to sell	Sold assets but usually to clear a debt at private providers.	Assets not sold to pay for treatment because there were other 'better' options
Sale of labor on farms	Preferred but not used due to drought	A possibility but drought limited its use	Unlikely for these households to use the strategy
Borrowing drugs	Preferred because they had no access to cash and were not required to pay back drugs	Not reported	Not reported
Sharing drugs	A common strategy when drugs are borrowed or bought	Common for households with many children	Reported when more than one child fell ill at the same time
Ignoring illness	A common strategy because they rarely had cash and access to other strategies was limited	Reported on two occasions because illnesses not perceived serious enough	Not reported
